# Measurement instruments for the core outcome set of congenital melanocytic naevi and an assessment of the measurement properties according to COSMIN: a systematic review

**DOI:** 10.1016/j.jpra.2022.11.003

**Published:** 2022-11-23

**Authors:** A.C. Fledderus, T. Boom, C.M. Legemate, C.M.A.M. van der Horst, P.I. Spuls

**Affiliations:** a Department of Plastic, Reconstructive and Hand Surgery, Amsterdam University Medical Center, University of Amsterdam, Meibergdreef 9, 1105 AZ Amsterdam, the Netherlands; b Department of Dermatology, Amsterdam Public Health, Amsterdam University Medical Center, Meibergdreef 9, 1105 AZ, Amsterdam, the Netherlands; c Department of Dermatology, Erasmus MC University Medical Center Rotterdam-Sophia Children’s Hospital-, Doctor Molewaterplein 40, 3015GD Rotterdam, the Netherlands.

**Keywords:** Congenital melanocytic naevi (CMN), Core outcome set (COS), Measurement instruments, Patient reported, Melanoma, Measurement properties

## Abstract

**Background:**

Congenital melanocytic naevi (CMN) can impact on patients’ lives due to their appearance and the risk they carry of neurological complications or melanoma development. The development of a core outcome set (COS) will allow standardised reporting and enable comparison of outcomes. This will help to improve guidelines. In previous research, relevant stakeholders reached a consensus over which core outcomes should be measured in any future care or research. The next step of the COS development is to select the appropriate measurement instruments.

**Aim:**

Step 1: to update a systematic review identifying all core outcomes and measurement instruments available for CMN. Step 2: to evaluate the measurement properties of the instruments for the core outcomes.

**Methods:**

This study was registered in PROSPERO and performed according to the PRISMA checklist. Step 1 includes a literature search in EMBASE (Ovid), PubMed and the Cochrane Library to identify core outcomes and instruments previously used in research of CMN. Step 2 yields a systematic search for studies on the measurement properties of instruments that were either developed or validated for CMN, including a methodological quality assessment following the COSMIN methodology.

**Results:**

Step 1 included twenty-nine studies. Step 2 yielded two studies, investigating two quality of life measurement instruments.

**Conclusion:**

Step 1 provided an overview of outcomes and instruments used for CMN. Step 2 showed that additional research on measurement properties is needed to evaluate which instruments can be used for the COS of CMN. This study informs the instrument selection and/or development of new instruments.

## Introduction

Congenital melanocytic nevi (CMN) are birthmarks present at birth or soon after birth. CMN are associated with an increased risk of melanoma, neurological complications and/or psychological burden due to their appearance^1^, ^2^, ^3^. Treatment of CMN is either conservative (watchful waiting including histology) or interventional (full thickness: excision, partial thickness: laser, curettage or dermabrasion). Outcomes measured to evaluate the treatment of CMN are heterogeneous in care and research, which impedes the comparison and pooling of these outcomes^4^. This complicates the guidance of optimal management policy.

The aim of the Outcomes for Congenital Melanocytic Naevi (OCOMEN) project is to develop a core outcome set (COS) for measuring the outcomes of all treatment options for medium, large and giant CMN for care and research^5^^,^^6^. A ‘COS’ is a consensus-derived minimum set of outcomes that should be measured and reported in all care and clinical trials of a certain health condition^7^^,^^8^. The use of a COS may enhance homogeneity in outcome and measurement instrument reporting in future studies and could therefore facilitate evidence synthesis for conservative and interventional treatment recommendation in the future.

In this study, we define ‘domains and outcomes’ as aspects of a disease that could be measured to evaluate different management strategies. ‘Domains’ are broader aspects of a disease, whereas ‘outcomes’ are defined as more precise aspects of a disease on a lower hierarchical level, like ‘presence of melanoma’ is an outcome of the domain ‘neoplasm’.

Patients included in the OCOMEN project are those presenting with either M1 (1.5–10 cm projected adult size (PAS)) on the face or M2 (>10–20 cm PAS) elsewhere, either single or multiple. The COS will be developed for international use in order to evaluate both interventional treatment and conservative treatment. In a recent consensus procedure, relevant stakeholders reached a consensus on the core domains and outcomes that need to be measured in the COS (Table 1)^5^^,^^6^^,^^9^. The next step in the development of the COS is to reach a consensus on how these domains must be measured (the core outcome measurement set (COMS)). The first step of developing the COMS is to identify all instruments previously used to measure core domains and outcomes and to evaluate the quality of the measurement properties of the instruments available for the core outcomes. A previous systematic review was performed summarizing all outcomes and measurement instruments used in research for CMN between 2006 and 2019 including sixty-three individual studies^4^. This study, as part of the OCOMEN project, aims to update this previously performed systematic review summarising all outcomes and their measurement instruments available for CMN. The second aim of this study is to critically appraise the measurement properties of all available measurement instruments that are developed and/or used in CMN patients, measuring the core outcomes.

**Table 1:** Core domains and outcomes of the COS of care and research.

Domains	Outcomes for the COS of care	Outcomes for the COS of research
1. Anatomy of skin	Size of CMN	Size of CMN
	Colour of the CMN	Colour of the CMN
	Texture of the CMN	Texture of the CMN
	Satellite nevi number	Satellite nevi number
2. Quality of life	Emotional distress	Emotional distress
3. Neoplasms	Presence of melanoma	Presence of melanoma
4. Nervous system	Neurological symptoms and signs	Neurological symptoms and signs
5. General adverse events	Wound problems of the CMN	Wound problems of the CMN
	Scar problems	Scar problems
6. Pathology		Molecular characteristics

## Methods

### This study consists of two steps

Step 1: A systematic review to identify and describe the outcomes and instruments used in previously published studies for CMN, as an update from a previously performed systematic review^4^. The previously systematic review included all outcomes and instruments used; the update only focusses on the outcomes of the COS and their instruments.
Step 2: A systematic review to evaluate the quality of the measurement instruments developed or validated for domains and outcomes of the COS of CMN.

Both these steps were registered in PROSPERO, registry number CRD42021238242, and reported according to the PRISMA checklist. The design of the systematic review was based on the guidelines of the Core Outcome Measures in Effectiveness Trials (COMET) initiative and the Cochrane Skin Group Core Outcomes Set Initiative (CS-COUSIN). The Consensus-based Standards for the Selection of Health Status Measurement Instruments (COSMIN) methodology and guidelines were used to critically appraise the measurement properties of instruments. The OCOMEN project was registered in the COMET initiative database.

Step 1: Identification and description of instruments used in previously published studies

### Search strategy, quality assessment and data extraction

This first step is an update of a previously performed systematic review in which a list of domains, outcomes and measurement instruments used in CMN research published between 2006 and 2019 were identified^4^. The search strategy used the current and previously performed systematic review was developed with the help of an information specialist (FE) and was performed in EMBASE (Ovid), PubMed and the Cochrane Library. The complete search strategy can be found in Appendix 1. The research for the current systematic review was performed between January 2019, which marked the end date of the previously performed systematic review^4^, and February 2021.

The same inclusion criteria from the previous systematic review were adopted for this study. We included all studies with ten or more patients that were written in English or Dutch. We excluded case reports, conference reports and books. Study selection was performed by two independent reviewers (ACF and TB), and disagreements were discussed with a third reviewer. Quality assessment of the included studies was performed independently by two researchers (ACF and TB) according to the level of evidence guidelines set by the Oxford Centre for Evidence-based Medicine^10^. Any disagreement regarding a study's level of evidence was resolved by discussion.

We extracted the following data: study characteristics (author, year, country, study design, intervention, number of subjects with CMN and classification system used for CMN), core domain, core outcomes and their measurement instruments. Unlike the previously performed review, we only extracted the core outcomes and the measurement instruments for the core outcomes. When diagnoses other than CMN were included in the studies, only data from CMN subjects was extracted. Data extraction was conducted independently by two reviewers (ACF and TB). Disagreements were resolved by discussion, or a third reviewer was consulted.

### Data synthesis

Data on domains, outcomes and measurement instruments were extracted. Descriptive statistics were used to calculate the frequency of outcomes. Measurement instruments were labelled as clinician reported or patient-reported outcome measurement instruments (PROMs).

Step 2: Evaluation of the quality of measurement instruments developed or validated for CMN

### Search and study selection

A search was performed in MEDLINE and EMBASE to identify development and validation studies of instruments for CMN that measured the core outcomes. It used the same controlled terms and words for the concepts of CMN that were used for the search strategy of Step 1 (Appendix 1), including a validated search filter for finding studies on measurement properties, developed by Terwee et al. (sensitive version, Appendix 2)^11^.

Only studies reporting on the evaluation of at least one measurement property of an instrument used or developed for CMN were included. The COSMIN taxonomy was used to select which of the following measurement properties of an instrument were evaluated: structural validity, internal consistency, reliability, hypotheses testing, cross-cultural validity and/or responsiveness^12^^,^^13^. We included both clinician reported and PROMs instruments including rating systems, questionnaires, medical devices or other instruments.

The following data were extracted independently by two reviewers (ACF and TB): study characteristics, patient characteristics, evaluated instruments, aspects of the measurement properties investigated and feasibility aspect of the instruments. Discrepancies were discussed with a third reviewer until a consensus had been reached.

### Evaluation of the methodological quality of the included studies

The COSMIN Risk of Bias checklist was used to evaluate methodological quality of the included studies^12^^,^^13^. Studies were stratified as having very good, adequate, doubtful or inadequate methodological quality.

### Assessment of measurement property results, best evidence synthesis and generating recommendations

Two authors (ACF and CML) independently rated the results of each study on a measurement property against the criteria for good measurement properties as either sufficient (+), insufficient (-) or indeterminate (?), as recommended by COSMIN^14^^,^^15^.

Results were summarized to produce an overall rating for each individual measurement property of every instrument. Next, the Grading of Recommendations, Assessment, Development and Evaluation (GRADE) approach was used to grade the quality of the evidence and thereby the trustworthiness of the results. A risk of bias (as determined using the COSMIN Risk of Bias checklist), the consistency of the study results on measurement properties across studies, and the sample size could all downgrade the evidence quality rating^14^.

Methods for generating recommendations for the measurement instruments of outcomes used for CMN were based on the methodological quality of the included studies and on the adequacy of an instrument. Four degrees of recommendation were assigned to the instruments included in this review (A-D) and adopted from previously performed studies^16^^,^^17^: category A, meets all requirements (positive rating for all boxes/measurement properties in the best evidence synthesis) and is recommended for use; B, meets two or more required quality items, but performance in all other required quality items is unclear, so the instrument has the potential to be recommended, depending on the results of further validation studies; C, exhibits low quality in at least one required quality criterion (≥1 rating of ‘minus’) and therefore is not recommended for further use; D, almost not validated, its performance in all or most relevant quality items is unclear, so further validation studies are needed.

## Results

Results Step 1: Identification and description of instruments used in previously published studies

### Search strategy, quality assessment and data extraction

**Figure 1:**
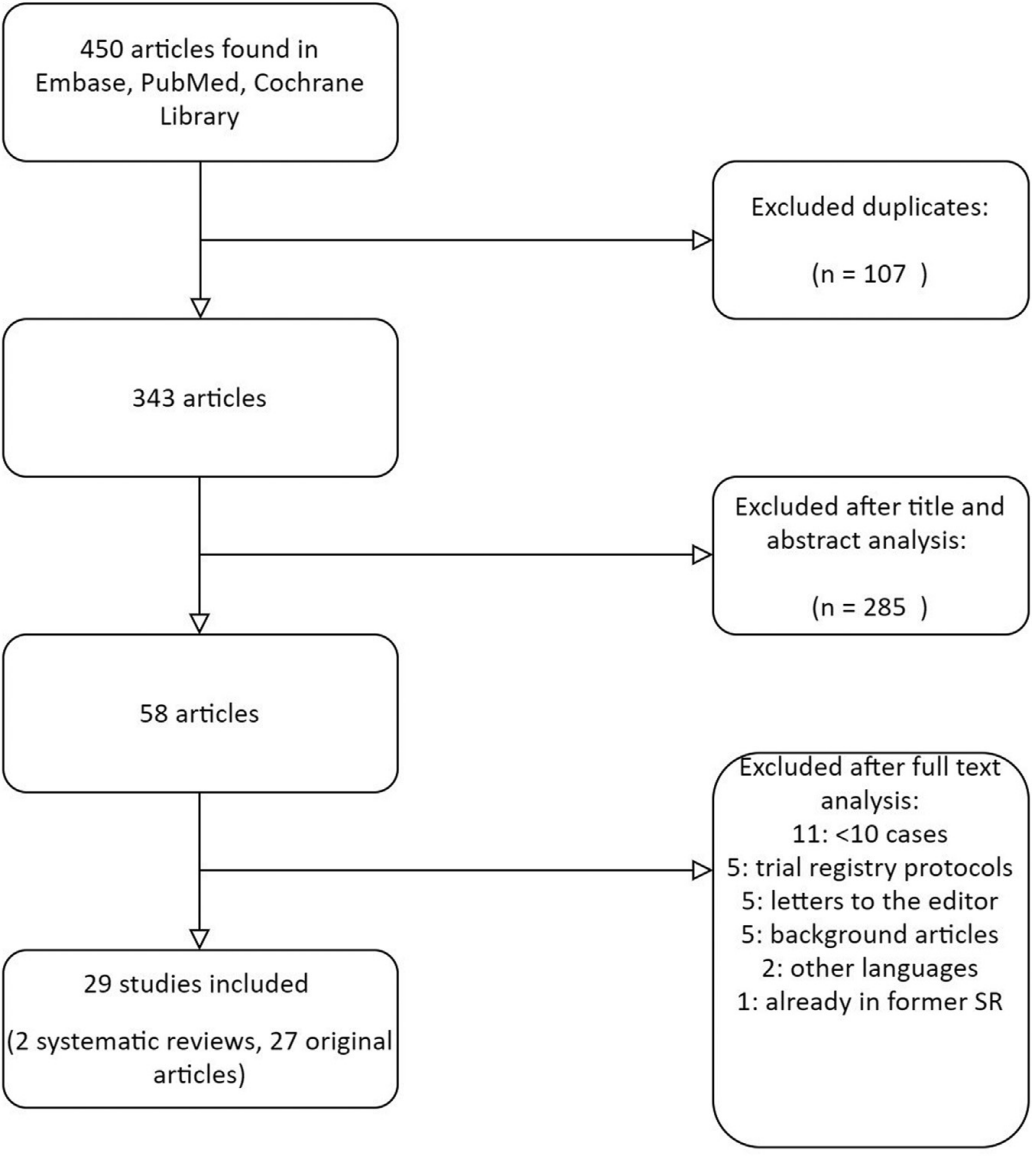
Search 1: Flow diagram.

The update from the previously performed systematic review yielded a total of 450 unique references after de-duplication. A total of 29 studies met the inclusion criteria, including 27 original studies with a total of 1938 patients and two systematic reviews. The selection procedure is illustrated in the flow chart of Figure 1.

Patient and CMN characteristics of the included studies are listed in Appendix 3. Most studies were conducted in Asia (45%), followed by Europe (35%) and the USA/Canada (10%). Two studies were conducted in the Middle East and one in Egypt. Thirteen studies had a prospective study design (45%). A total of 12 studies were retrospective (41%). Two studies were cross-sectional (7%). Two systematic reviews (7%) were detected with a total of 35 studies.

Similar to the previously performed systematic review, the quality of the studies included in the update was generally low. Most studies (55%) were rated as level 3 evidence (low evidence). All other studies, 13 in total (44%), were rated as level 4 (very low evidence). The level of evidence was mainly low because of small patient groups, the absence of control groups and retrospective study designs.

The number of included patients ranged from 15 to 293 CMN patients in the update, and the female to male ratio was 1.35:1. The mean patient age was 15.2 years (range 0–73 years) mentioned in 16 out of 29 studies.

We found different classification systems used for CMN, equally to the previous systematic review. For location, most studies reported a particular part of the body, but body parts were sometimes classified together. Size was defined in the following ways: the diameter in centimetres in PAS (11 studies) and the percentage of the total body surface area (TBSA) (four studies). The classification of Krengel et al. was used in five studies. Two studies used the ‘6B rule’ to classify the location of giant CMN. Twelve studies did not define size according to a certain classification system.

### Data synthesis

Table 2 shows the frequency of the core outcomes reported in the 29 studies of the update and their frequency in the sixty-three studies performed in the previous systematic review^4^. Table 3 shows the measurement instruments used to measure the core outcomes found in the previously performed systematic review and the update, including information on the instrument, the target population, and whether it was a PROM or clinician reported.

**Table 2:** Frequency of outcomes 75, 76, 77, 78, 79, 80, 81.

Core Domain	Core outcome used in research	Reported previous SR		Reported update SR	
		N	%	n	%
Anatomy of skin	Size of CMN	1/63	2	3/29	10
	Colour of the CMN	31/63	49	5/29	17
	Texture of the CMN	21/63	33	3/29	10
	Satellite nevi number	10/63	16	0/29	0
Quality of life	Emotional distress	6/63	10	7/29	24
Neoplasm	Presence of melanoma	21/63	33	14/29	48
Nervous system	Neurological symptoms and signs	11/63	17	5/29	17
General adverse events	Wound problems of the CMN	32/63	51	11/29	38
	Scar problems	10/63	16	12/29	41
Pathology	Molecular characteristics	2/63	3	7/29	26

n: number of studies reporting the outcome; SR: systematic review

**Table 3:** Measurement instruments.

Measurement instrument	Outcome measured⁎	Description of instrument	Target population	CR/PROM
Anatomy of the skin (domain)				
Digital assessment of length in cm^45^	CMN lesion size and postoperative scar size	Digital measurement of the size of the lesion in two dimensions	S, M, L CMN Age NR	CR
Length in cm measured by a ruler^46^	Long diameter of nevi	To measure the long diameter in centimetres by using a ruler.	Size NR Age range: 10-103m	CR
Rating system, self- or proxy-reported^47^	Percentage of nevusremoval	Patients or caregivers were asked to rate removal percentages (<10%, 10–25%, >25–50%, >50–75%, >75%)	S, M, L, G CMN Mean age: 17.5 y [self] and 6.3 y [proxy]	PROM
Tracing on transparent film (area of resection in cm^2^)^46^	Area of nevi before resection in cm^2^	To measure the area, trace the total nevus area onto a transparent film and then transfer it to paper divided into millimetres	Size NR Age range: 10-103m	CR
L*a*b-colour space model (CIE-LAB) on clinical photos^48^	Lightening / colour change of CMN	A program using mathematical descriptions of all perceivable colours in three dimensions	S, M, L, G CMN Age range: 0-17.4y	CR
Kilmer & Lee 5 point lightening scale^49^^,^^50^	Lightening of CMN	A 5-step scale to measure lightening of CMN colour after laser treatment: poor, fair, good, excellent and clear	S, M CMN Median age: 9 m Age range: 0-28 y	CR
Zaal & van der Horst 7-point repigmentaion scale^51^	Repigmentation	A 10-step scale to measure repigmentation after treatment: 1-4 mild, 5-7 moderate and 8-10 severe.	G CMN Age range: 0.4-36 y	
Self-made questionnaire by Kinsler et al.^52^	Colour lightening or darkening, hairiness, lumpiness, new CMN in unaffected skin at the edge of the treated area and development and number of new satellite lesions.	A questionnaire to measure the changes in the CMN appearance and the development of new satellites	S, M, L, G CMN Mean ag: 2.9 y	PROM
Estimation by specialist^53^	Hypopigmentation, hyperpigmentation, repigmentation, infection, erythema, scarring,	Reviewing of clinical photographs by clinician	Size NR Age range: 7-25 y	CR
Evaluation by specialist blinded to study^54^	Reduction pigmentation after treatment	Independent third party reviewed clinical photographs	Size NR Mean age: 12 y	CR
Investigator's Global Assessment (IGA) score for skin appearance^55^	Pigment clearance, erythema, hypopigmentation, hypertrophic scaring and texture irregularity	A 7-point scale to assess the improvement of clinical outcomes before and after intervention: 1 worsened - 7 total improvement	Size NR Mean age 13.4 y	CR
Quality of life				
Paediatric Outcomes Data Collection Instrument (PODCI)^56^	Physical functioning, mobility, sports, pain/comfort and happiness	To estimate functional health outcomes, musculoskeletal health, and QoL. It has been extensively reported in the orthopaedic surgery literature. Number of items: 86 Scoring method: Likert-scale, scores range from 0–3 for some items and 0–6 for others Total score range: 0 worse -100 best	Size/age NR	PROM
Children's Dermatology Life Quality Index (CDLQI)^19^^,^^57^^,^^58^	Skin discomfort, emotional, social and physical functioning, teasing/ bullying/ asking questions, sleep, effect of treatment on QoL	To assess proxy- and self-reported skin-related QoL No of items: 10 questions Scoring methods: 4-Point Likert scale Total score range: sum score, range, 30 best - 0 worst	S, M, L, G CMN Age range: 4-18 y	PROM
Pediatric Quality of Life Inventory 4.0 (PedsQol)^18^	Health-related QoL, Emotional functioning, Social functioning	To assess self- and proxy-reported Health-related QoL Number of items: 21 Scoring method: 5-point Likert scale Total score range: 0 never - 4 almost always	S, M, L, G CMN Mean age: 6.3 y	PROM
Strengths and Difficulties Questionnaire (SDQ)^18^	Psychological adjustment, emotional conduct, hyperactivity, inattention	To assess self- and proxy-reported emotional and behavioural problems Number of items: 25 Scoring method: 3-options, not true – somewhat true – certainly true Total score range: sum score 0 - 40	S, M, L, G Mean age: 6.3 y	PROM
Post-Traumatic Stress Disorder Semi structured Interview (PTSDSSI)^59^	Anxiety, depression, withdrawal, somatic complaints, attention problems, thought problems, social problems, rule-breaking behaviour and aggressive behaviour	To assess the frequency of PTSS Number of items: 29 Scoring method: mixed response no 0, sometimes 1, yes 2	Size NR Mean age: 4.2 y	PROM
Teacher Report Form^60^	Academic competence, adaptive functioning, inattention, hyperactivity, impulsivity, social problems, thought problems, anxious and depressed	To rate the child's behavioural competence and behavioural/emotional problems Number of items: 113 Scoring method: 3-point Likert scale 0 Not True, 1 Somewhat or Sometimes True, and 2 Very True or Often True and fill-in blanks questions	L CMN Mean age: 12.6 y	PROM
Estimation by parents^61^	Estimation by parents of global QoL	Global QoL	S, M, L, G CMN Age NR	
Neoplasms (Cancer)				
Questionnaire for presence of malignancy (proxy report)^18^^,^^19^^,^^47^	Presence of Melanoma	To indicate the patients’ health status concerning chronic diseases such as the presence of melanoma.	S, M, L, G CMN Various ages	PROM
Histopathological biopsy (unspecified assessor)^62^^,^^63^	Presence of Melanoma	To assess histologically for melanoma presence in biopsied CMN lesions	S, M, L, G CMN Various ages	CR
Clinical photos and Dermascopy^64^	Presence of Melanoma	A non-invasive and in vivo diagnostic tool to visualize subtle clinical patterns of skin structures invisible to the unaided eye.	Size NR Mean age: 39.2 y	CR
Nervous system				
Questionnaire (proxy report), presence of neurological problems^18^^,^^19^^,^^47^	Neurological symptoms and signs	To indicate the patients’ health status concerning chronic diseases such as the presence of neurological problems.	S, M, L, G CMN Various ages	PROM
EEG, and classification criteria of ILAE^65^	Focal epilepsy	To assess the diagnosis of focal epilepsy, an EEG was performed and was classified according to the ILAE criteria.	G CMN, Median age: 5 m	CR
Developmental milestones assessment^65^	Cognitive developmental delay	To distinguish cognitive development as normal or delayed, the developmental milestones in children were used.	G CMN, Median age: 5 m	CR
Physical evaluation by physician^66^	Neurological symptoms and signs	Assessment of neurological symptoms and signs by a clinician	M CMN Age range: 9-43 y	CR
Adverse events				
Clinical photographs and visual assessment (surgeons)^45^^,^^53^^,^^67^	Wound problems of the CMN Scar problems	Based on photographs, the scars and wound problems were visually assessed by clinicians	S, M, L CMN and ‘kissing naevus’ Various ages	CR
Vancouver Scar Scale (VSS) (3 independent evaluators)^68^	Skar appearance, skar pigmentation, skar height/thickness, skar pliability, and skar vascularity	A tool for scar assessment, with the highest score indicating the worst scar formation and 0 suggesting the best outcome 0 best outcome – 4 worst outcome)	M CMN Mean age: 20.4 y	CR
Investigator's Global Assessment (IGA) score for skin appearance^55^	Pigment clearance, erythema, hypopigmentation, hypertrophic scaring and texture irregularity	A 7-point scale to assess improvement of clinical outcomes before and after intervention: 1 worsened - 7 total improvement	Size NR Mean age: 13.4 y	CR
Own assessment (self/proxy report)^18^^,^^47^	Healing issues or infections	Patients or parents could indicate that if they had wound healing problems through a questionnaire	S, M, L, G CMN Various ages	PROM
Patient and Observer Scar Assessment Scale (POSAS-score)^45^^,^^47^^,^^51^^,^^69^	Scar appearance	Observer and patient scale Number of items: 6 Scoring method: 10-step score, 10 worst imaginable scar Total score range: 6 reflects normal skin – 60 the worst imaginable scar	S, M, L, G CMN Various ages	CR/ PROM
Physical examination^70^	Infection, hypertrophic or atrophic scarring	To assess the occurrence of adverse events, a physical examination was performed during follow-up.		CR
Self-made questionnaire by August et al.^66^	General adverse events	Participants could indicate if they had any side effects from the treatment? Rating: 1-10 (10 being worst)	M CMN Age range: 9-43 y	CR
Pathology				
Electrochemiluminescence immunoassay^71^	Molecular characteristic	To determine S-100B protein concentrations in peripheral blood in a blinded manner	M, L, G CMN Mean age: 5.7 y	CR
Phosphokinase-array^72^	Molecular characteristic	To analyse the expression of effector proteins of the MAPK/Akt signalling pathways	L, G CMN Median age 8 m	CR
PCR - MC1R screening blood/saliva samples^73^	Molecular characteristic	To amplify two overlapping fragments of the MC1R-coding region in blood and saliva samples	M, L, G CMN Mean age: 16.8 y	CR
Sanger sequencing^48^	Molecular characteristic	Germline MC1R-genotyping was undertaken on leucocyte DNA	S, M, L, G CMN Age range: 0.0-17.2 y	CR
Immunohistochemistry^63^	Molecular characteristic	To assess the proliferative indices in Giant CMN lesions by using proliferation markers (Ki67, Melan-Am S-100, HMHB-45 and SOX-10)	G CMN Median age: 6 y	CR
Single-base extension SNaPshot assay PCR^74^	Molecular characteristic	To analyse recurrent point mutation in KRAS codons G12, G13 and Q61; NRAS codons G12, G13 and Q61; HRAS codons G12, G13 and Q61; GNAQ exon 5; and BRAF codon V600 in proliferative noduli tissue	S, M, L, G CMN Age range: 0-84 y	CR

⁎ The specific core outcome of the core domain in underlined. CR: clinician reported, PROM: patient-reported outcome measure, S: small, M: medium, L: large, G: giant, NR: not reported, y: years, m: months, QoL: quality of life

Results Step 2: Evaluation of the quality of measurement instruments developed or validated for CMN

### Search and study selection

**Figure 2:**
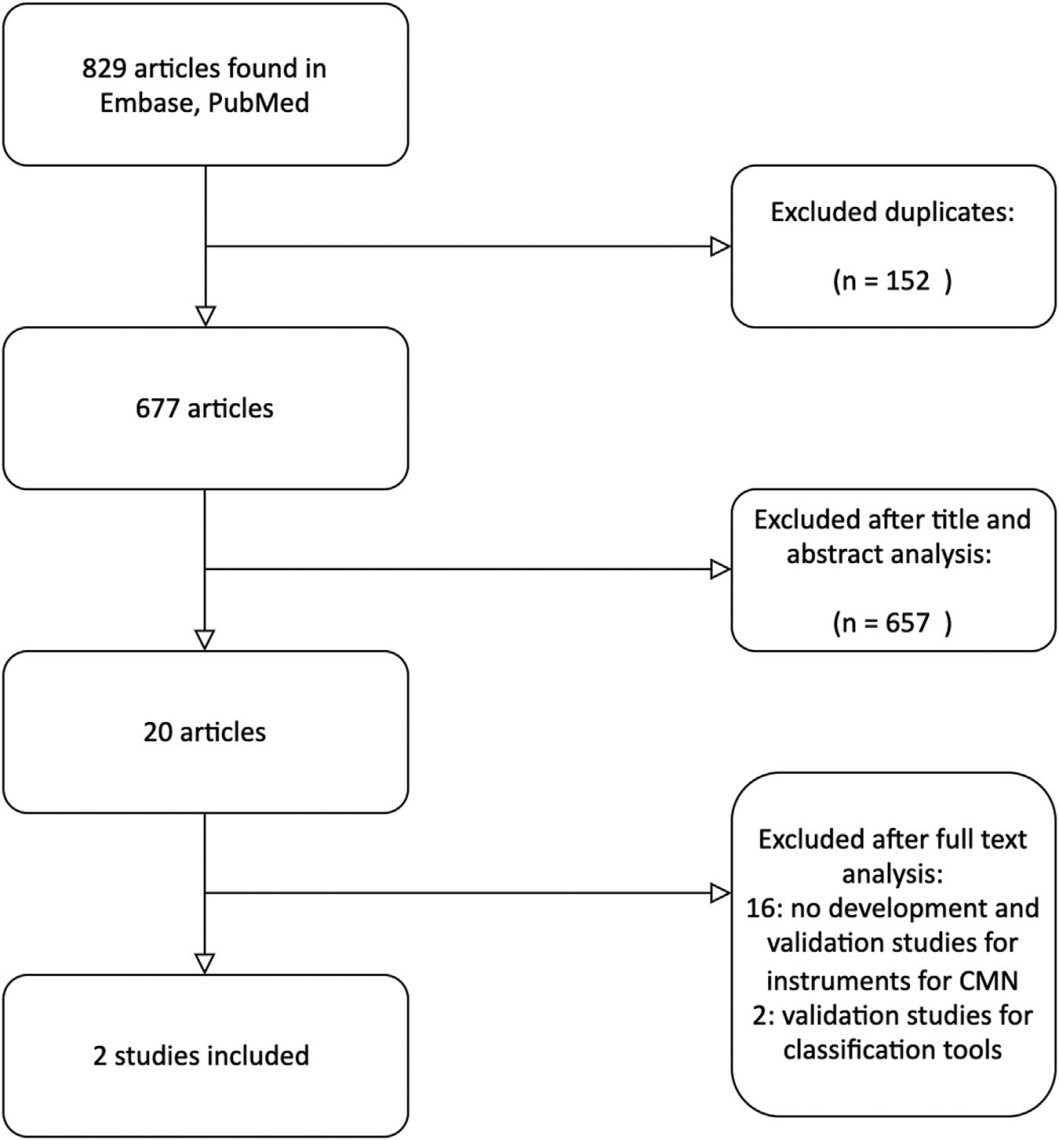
Search 2: Flow diagram.

The search provided 677 unique studies; Figure 2 shows the flow diagram of the study selection. Two studies met our inclusion criteria, with both evaluating one measurement property, internal consistency, of an instrument measuring the domain ‘quality of life’^18^^,^^19^.

We did not find any development studies. Besides ‘quality of life,’ there were no studies available for instruments measuring the other core domains and outcomes developed or validated for the CMN population. Moreover, no clinician reported instruments rating systems, medical devices or other instruments were developed or validated for CMN.

### Evaluation of the methodological quality of the included studies

Both studies had scored a ‘very good’ for their methodological quality regarding the measurement property they assessed (Appendix 4).

### Evaluation of the quality of the measurement properties, evidence synthesis and generating recommendations

The included studies evaluated the measurement property ‘internal consistency’ of the Paediatric Quality of Life Inventory (PedsQol) and the Children's Dermatology Life Quality Index (CDLQI) in order to measure the domain ‘quality of life,’ including the outcome ‘emotional distress’^18^^,^^19^. The following measurement properties were not evaluated: structural validity, reliability, hypotheses testing, cross-cultural validity and/or responsiveness. We did not find any study evaluating these measurement properties in other instruments used for the CMN population.

Masnari et al. studied internal consistency of the PedsQol. They recruited their patients worldwide and included 235 children with a mean age of 6.3 years and a mean TBSA score of 13.14 percent. About half of the included children did not have any surgery to remove the CMN.

Neuhaus et al. studied internal consistency of the CDLQI and recruited their patients worldwide as well. They included 163 patients. The mean age of children in their proxy-report group (4-18 years) was 9.3 years and in the self-report group (14-18 years) was 16.3 years. They had a mean TBSA score of 13.6 and 16.1, respectively. More than half of the patients underwent partial removal of their CMN.

Table 4 shows the rating of the results and level of evidence.

**Table 4:** Study characteristics and rating of internal consistency.

Measurement instrument	Sample size	Results (Cronbach's α)	COSMIN Risk of Bias score^2^	Level of evidence^3^	Rating of results^6^
PedsQol^18^					
1-12m	31	0.76 - 0.94^1^	Very good	Low^4^	?^7^
13-24m	32	0.72 - 0.91^1^	Very good	Low^4^	?^7^
2-18y	170	0.53 - 0.94^1^	Very good	Moderate^5^	?^7^
CDLQI^19^					
Proxy	135	0.83	Very good	High	?^7^
Self- report	28	0.87	Very good	Low^4^	?^7^

1: Range of cronbachs's α: for each item, cronbach's α was measured separately.

2: Based on the COSMIN risk of bias tool (Appendix 4).

3: After application of the GRADE approach.

4: Downgraded because of the sample size.

5: Downgraded because of the indirectness, as the exact sample size of the size of the 5–18 years and < 5 years groups is not reported.

6: Rating of results was either sufficient (+), insufficient (−) or indeterminate (?).

7: Rated as indeterminate due to the absence of evidence for sufficient structural validity.

Despite most Cronbach alpha item scores being >0.7, all ratings were scored as indeterminate due to the absence of “at least low evidence for sufficient structural validity”, which is a requirement for a sufficient rating for internal consistency. Table 5 shows the feasibility aspects of these instruments. The best evidence synthesis is shown in Table 6. As only the internal constancy of these questionnaires had been evaluated, they received recommendation D, indicating that they were almost not validated. Its performance in all or most relevant quality items is unclear; further validation studies are needed.

**Table 5:** Aspects of feasibility.

Instrument	Available for ages	Available translations	Completion time	Licensing/costs
CDLQI Self- and proxy-reported	4-12 years proxy- and self-reported Adult version available (DLQI)	115 Languages	2 min	Free for clinicians, free for non-academic research (not funded externally); external funded trial fees dependent on sample size
Self- and proxy-PedsQoL	2-18 years proxy- and self-reported	176 translations	4 min	The costs are determined based on the type of research, the source of funding for the research and the sample size.

**Table 6:** Best evidence synthesis and recommendations.

Evaluated measurement properties according to the COSMIN taxonomy^12^	PedsQol	CDLQI
	Masnari et al. (2019)^18^	Neuhaus et al. (2020)^19^
Internal consistency	?	?
Reliability	NA	NA
Measurement error	NA	NA
Content validity	NA	NA
Structural validity	NA	NA
Hypotheses testing	NA	NA
Cross-cultural validity	NA	NA
Responsiveness	NA	NA
Recommendation	Category D	Category D

For each measurement property, the methodological quality of the study is reported as sufficient (+), insufficient (−)or indeterminate (?), *NA* not available (analysis was not performed for this measurement property).

Recommendations: category A, meets all requirements (positive rating for all boxes in the best evidence synthesis) and is recommended for use; B, meets two or more required quality items, but performance in all other required quality items is unclear, so that the instrument has the potential to be recommended, depending on the results of further validation studies; C, low quality in at least one required quality criteria (≥1 rating of ‘minus’) and therefore is not recommended to be used anymore; D, almost not validated. Its performance in all or most relevant quality items is unclear; further validation studies are needed.

## Discussion

This study is the first step of selecting the core measurement instruments for the COS of CMN. We showed a systematic overview of the instruments used to measure core outcomes for CMN published in addition to a previously performed study^4^. In addition, studies on measurement properties of instruments used for the CMN population were evaluated. We found a wide heterogeneity in outcomes and measurement instruments in the included studies, and there were no studies reporting all core outcomes. We showed that research on measurement properties of these instruments is limited. Therefore, none of the instruments could be recommended based on the quality of their measurement properties, and further validation studies are needed.

Research on CMN is growing; this current update included twenty-nine studies published in a period of two years, while the previously performed systematic review includes sixty-three studies in a period of twelve years^4^. Uniformity is therefore of upmost importance to enable combination and comparison of studies. However, heterogeneity in outcomes still exist, highlighting the importance of a COS. Besides heterogeneity in outcomes, we found heterogeneity in CMN classifications as well. To enhance uniformity in CMN care and research, we recommend using the consensus derived, internationally used classification developed by Krengel et al.^20^ and qualified (the “6B”^21^ and “biker glove” distributions^22^) for the CMN location.

Relevant stakeholders should reach consensus over which instruments should be validated for CMN. In this process, the feasibility of instruments should also be considered as well; instruments should be easy and quick to use and should be low-cost or free of charges. Similar systematic reviews investigating the measurement properties according to the COSMIN checklist are available for diseases similar to CMN such as vitiligo, vascular malformations, capillary malformation and burn scars^17^^,^^23^, ^24^, ^25^. Although these studies also revealed a low quality of measurement instruments validated for their particular patient population, some of their recommendations may inform which instruments should be validated for CMN.

The domain ‘anatomy of the skin’ or ‘skin appearance’ is often measured by disease-specific measurement instruments, a probable result of the unique manifestations of every skin disease. For CMN, we found both objective instruments, such as L*a*b* colour-space model (CIE-LAB) measurements, as well as subjective rating systems (Table 3). The systematic reviews of similar anomalies revealed that ‘skin appearance’ is generally measured by questionnaires or rating systems completed by both clinicians and patients. These types of instruments are often low-cost and quick and easy to use. For vitiligo, the most effective instrument that measures the size of a lesion was the disease specific (Self-Assessment) Vitiligo Extent Score ((SA)-VES)^26^. For capillary malformation, there were only low-quality clinician reported rating systems available^25^. None of these rating systems were developed by asking patients (or their parents) to determine which outcomes are important to them^25^. The systematic review for vascular malformations also showed low-quality rating systems^17^. Therefore, a new PROM questionnaire is now in development; the Outcome Measures for Vascular Malformations (OVAMA) questionnaire^27^. For burn scars, both PROMs, clinician reported rating systems and objective measurement instruments are available^28^. For instance, objective instruments to measure the colour of burn scars include the following: reflectance spectroscopy (colorimetry/spectrophotometry), laser imaging or computerized analysis of digital photographs^29^.

Various questionnaires are available to measure the domain ‘quality of life’, including the outcome ‘emotional distress’, in patients with a skin disease. To measure health-related ‘quality of life’, disease-specific instruments and generic instruments are available. In addition, for skin conditions, dermatology specific questionnaires are available^30^. Disease-specific instruments measure the impact of a specific condition on the different aspects of ‘quality of life’, while generic instruments measure the overall ‘quality of life’ of a subject, allowing comparisons between a group of patients with a certain disease and their peers of the general populations. The systematic review evaluating ‘quality of life’ instruments for burn scars showed that burn scar specific instruments have the best measurement properties^24^.

No disease-specific questionnaires are available for CMN. Rare diseases may be best measured with a generic ‘quality of life’ measurement instrument, as the development of a high-quality disease-specific instrument is hindered by the limited number of subjects to validate the instrument. An existing generic instrument may be the best option for CMN, as there are various generic quality of life PROMs available. The systematic review for capillary malformations provisionally recommends the PROMs Perceived Stress Questionnaire (PSQ) or the DLQI. The DLQI was proposed by the vitiligo group as well^23^. The systematic review for vascularity malformations states that the Short Form-36 (for adults) and PedsQol (for children) seem to be the most appropriate generic instrument^17^. However, this same research group showed in a subsequent study that these questionnaires do not sufficiently measure effectiveness, i.e., change in the ‘quality of life’ before and after treatment. They therefore advise using Patient-Reported Outcomes Measurement Information System (PROMIS)^27^^,^^31^. The use of PROMIS is advised for rare diseases and may be suitable to use for CMN^32^, ^33^, ^34^. PROMIS consists of item banks for every subdomain of ‘quality of life,’ which have been extensively validated in large populations. An item bank is a large set of questions for multiple ‘quality of life’ outcomes. These item banks are available in short form and with computer adaptive testing. With computer adaptive testing, the most relevant questions for an individual will be asked based on their previous answers. This decreases the number of questions and causes accurate and person-centred outcomes. In contrast to other generic instruments, PROMIS facilitates the measurement of the outcome ‘emotional distress’ without measuring the outcomes ‘social and physical functioning'.

For measuring the domain ‘neoplasm,’ a panel of stakeholders agreed that the core outcome ‘presence of melanoma’ should always be measured in care and research. In this study, we found that the ‘presence of melanoma’ to be measured by self-/proxy-report of patients or their parents through online questionnaires or by pathological confirmations. In future research, a consensus should be reached regarding whether melanoma should be confirmed by pathology for all research or if an anamnesis of patients or parents is sufficient for survey studies.

The domain ‘neurology’ is defined by the outcome ‘neurological symptoms and signs’. A consensus procedure with international stakeholders should be held to decide how neurological symptoms and signs should be measured. For instance, a questionnaire screening for the most common symptoms or signs could be used and/or stakeholders could decide that neurological examinations should be performed as a standard by, for example, a neurologist or paediatrician. None of the studies included in this study or the previously performed systematic review used a questionnaire for specific symptoms and signs of CMN patients^4^. Questionnaires to measure developmental delay or epilepsy are available for clinicians and for patients^35^, ^36^, ^37^, ^38^. Questionnaires to measure general neurology disorders are available and are frequently developed for patients in low- and mid-income countries^39^, ^40^, ^41^. If relevant stakeholders decide that a neurological questionnaire should be used for the COS, future research should assess the accuracy and feasibility of the questionnaires for neurological involvement in CMN patients or decide to develop a CMN-specific instrument.

The domain ‘general adverse event’ includes the core outcomes ‘wound problems of the CMN’ and ‘scar problems’. Classifications such as The Common Terminology Criteria for Adverse Events (CTCAE), the Medical Dictionary for Regulatory Activities (MedDRA) or the Clavien-Dindo Classification can be consulted to classify the severity or define the adverse events. A consensus should be reached over which classification should be used to report adverse events. For the outcome ‘scar problems,’ the Patient and Observer Scar Assessment Scale (POSAS) is used in four CMN studies. A new version of the POSAS is currently being developed, in which the patients’ opinion on scar appearance is implemented. A consensus with international stakeholders should be reached over which standard instrument and classification system should be used to report adverse events.

The importance of the outcome ‘molecular characteristics’ of the domain ‘pathology’ is growing in the research of CMN. A quarter of the studies included in this systematic review measured this outcome. Increasing knowledge regarding molecular characteristics of CMN could help in the future to estimate the risk of melanoma or neurological complications^42^. Moreover, new pharmacological therapies may be developed that could be offered to patients with a certain DNA mutation^43^^,^^44^. We showed that various molecular characteristics are reported in the literature. For now, alongside all relevant stakeholders, we have decided that all molecular characteristics that are already measured for care purpose should be standard documented in research of CMN in a standardised manner.

## Strengths and limitations

We systemically reviewed the availability and quality of measurement instruments of CMN according to the COMET, CS-COUSIN and COSMIN guidelines. We included a broad range of studies on CMN, including both outcomes and instruments for studies of intervention treatment and watchful waiting. A limitation could be that we only included studies written in English or Dutch; however, there is a wide geographical spread in the included publications. Because of the heterogeneity in the classification of CMN, we could not describe differences between measurement instruments used for different CMN size or location (visible/non-visible) categories.

## Future perspectives

This systematic review was the first step of developing the COMS of the COS of medium-to-giant CMN care and research. Relevant stakeholders should reach a consensus over which measurement instruments should be used for the domains and outcomes of CMN. Firstly, relevant stakeholders should decide whether every domain and outcome should be clinician and/or patient reported and if questionnaires, rating systems, clinical devices or other instruments are needed. In addition, they should consider the feasibility of an instrument. Secondly, relevant stakeholders should decide which measurement instruments should be developed or validated for the CMN patient population. This study informs the instrument selection and/or the development of new instruments.

## Declaration of Competing Interest

The authors have no other financial or personal relationships relevant to this study to disclose.

## Appendix Supplementary materials

Image, application 1
