# Supplementary material for: Measurement instruments for the core outcome set of congenital melanocytic naevi and an assessment of the measurement properties according to COSMIN: a systematic review
Source: JPRAS Open. 2022 Nov 23;35:58–75. doi: 10.1016/j.jpra.2022.11.003 (PMC9860390; doi:10.1016/j.jpra.2022.11.003)
Supplement: Supplementary file 1 [file mmc1.docx]

**Supporting materials**

**Appendix 1: Search 1**

**Pubmed:**

("Nevus"[Mesh] OR nevus OR nevi OR naevus OR naevi OR birthmark*)

AND

(congenital* OR bathing trunk* OR garment OR giant OR tierfell* OR gigantic OR inborn OR hereditary OR newborn OR "congenital" [Subheading])

NOT

(connective tissue[tiab] OR anaemicus[tiab] OR elasticus[tiab] OR inelasticus[tiab] OR depigmentosus[tiab] OR mucinosis[tiab] OR lipomatosus[tiab] OR sebaceus[tiab] OR blue[tiab] OR comedonicus[tiab] OR spindle[tiab] OR sponge[tiab] OR woolly[tiab] OR spilus[tiab] OR spider[tiab] OR flammeus[tiab] OR Jadassohn[tiab] OR Ota[tiab] OR Becker[tiab] OR Sutton[tiab] OR Unna[tiab] OR neurofibromatosis[tiab] OR pancreas*[tiab] OR placenta[tiab])

NOT

("Case Reports" [Publication Type] OR case report*)

**Embase(Ovid):**

| **#** | **Searches** |
| --- | --- |
| 1 | exp nevus/ or (nevus or nevi or naevus or naevi or birthmark*).ti,ab,kw. |
| 2 | (congenital* or bathing trunk* or garment or giant or tierfell* or gigantic or inborn or hereditary or newborn).ti,ab,kw. or cn.fs. |
| 3 | 1 and 2 |
| 4 | (connective tissue or anaemicus or elasticus or inelasticus or depigmentosus or mucinosis or lipomatosus or sebaceus or blue or comedonicus or spindle or sponge or woolly or spilus or spider or flammeus or Jadassohn or Ota or Becker or Sutton or Unna or neurofibromatosis or pancreas* or placenta).ti,ab,kw. |
| 5 | 3 not 4 |
| 6 | limit 5 to conference abstract status |
| 7 | 5 not 6 |
| 8 | case report/ or case report*.ti,ab,kw. |
| 9 | 7 not 8 |

**Cochrane Library:**

ID Search Hits

#1 nevus or nevi or naevus or naevi or birthmark*:ti,ab,kw (Word variations have been searched)

#2 congenital* or bathing trunk* or garment or giant or tierfell* or gigantic or inborn or hereditary or newborn:ti,ab,kw (Word variations have been searched)

**Appendix 2: COSMIN filter**

**COSMIN sensitive search filter for measurement properties**

(instrumentation[sh] OR methods[sh] OR Validation Studies[pt] OR Comparative Study[pt] OR “psychometrics”[MeSH] OR psychometr*[tiab] OR clinimetr*[tw] OR clinometr*[tw] OR “outcome assessment (health care)”[MeSH] OR outcome assessment[tiab] OR outcome measure*[tw] OR “observer variation”[MeSH] OR observer variation[tiab] OR “Health Status Indicators”[Mesh] OR “reproducibility of results”[MeSH] OR reproducib*[tiab] OR “discriminant analysis”[MeSH] OR reliab*[tiab] OR unreliab*[tiab] OR valid*[tiab] OR coefficient[tiab] OR homogeneity[tiab] OR homogeneous[tiab] OR “internal consistency”[tiab] OR (cronbach*[tiab] AND (alpha[tiab] OR alphas[tiab])) OR (item[tiab] AND (correlation*[tiab] OR selection*[tiab] OR reduction*[tiab])) OR agreement[tiab] OR precision[tiab] OR imprecision[tiab] OR “precise values”[tiab] OR test–retest[tiab] OR (test[tiab] AND retest[tiab]) OR (reliab*[tiab] AND (test[tiab] OR retest[tiab])) OR stability[tiab] OR interrater[tiab] OR inter-rater[tiab] OR intrarater[tiab] OR intra-rater[tiab] OR intertester[tiab] OR inter-tester[tiab] OR intratester[tiab] OR intra-tester[tiab] OR interobserver[tiab] OR inter-observer[tiab] OR intraobserver[tiab] OR intra-observer[tiab] OR intertechnician[tiab] OR inter-technician[tiab] OR intratechnician[tiab] OR intra-technician[tiab] OR interexaminer[tiab] OR inter-examiner[tiab] OR intraexaminer[tiab] OR intra-examiner[tiab] OR interassay[tiab] OR inter-assay[tiab] OR intraassay[tiab] OR intra-assay[tiab] OR interindividual[tiab] OR inter-individual[tiab] OR intraindividual[tiab] OR intra-individual[tiab] OR interparticipant[tiab] OR inter-participant[tiab] OR intraparticipant[tiab] OR intra-participant[tiab] OR kappa[tiab] OR kappa’s[tiab] OR kappas[tiab] OR repeatab*[tiab] OR ((replicab*[tiab] OR repeated[tiab]) AND (measure[tiab] OR measures[tiab] OR findings[tiab] OR result[tiab] OR results[tiab] OR test[tiab] OR tests[tiab])) OR generaliza*[tiab] OR generalisa*[tiab] OR concordance[tiab] OR (intraclass[tiab] AND correlation*[tiab]) OR discriminative[tiab] OR “known group”[tiab] OR factor analysis[tiab] OR factor analyses[tiab] OR dimension*[tiab] OR subscale*[tiab] OR (multitrait[tiab] AND scaling[tiab] AND (analysis[tiab] OR analyses[tiab])) OR item discriminant[tiab] OR interscale correlation*[tiab] OR error[tiab] OR errors[tiab] OR “individual variability”[tiab] OR (variability[tiab] AND (analysis[tiab] OR values[tiab])) OR (uncertainty[tiab] AND (measurement[tiab] OR measuring[tiab])) OR “standard error of measurement”[tiab] OR sensitiv*[tiab] OR responsive*[tiab] OR ((minimal[tiab] OR minimally[tiab] OR clinical[tiab] OR clinically[tiab]) AND (important[tiab] OR significant[tiab] OR detectable[tiab]) AND (change[tiab] OR difference[tiab])) OR (small*[tiab] AND (real[tiab] OR detectable[tiab]) AND (change[tiab] OR difference[tiab])) OR meaningful change[tiab] OR “ceiling effect”[tiab] OR “floor effect”[tiab] OR “Item response model”[tiab] OR IRT[tiab] OR Rasch[tiab] OR “Differential item functioning”[tiab] OR DIF[tiab] OR “computer adaptive testing”[tiab] OR “item bank”[tiab] OR “cross-cultural equivalence”[tiab])

**COSMIN FILTER adapted for EMBASE(Ovid)**

methodology/ or exp health status indicator/ or Sickness Impact Profile/ or clinical assessment/ or clinical assessment tool/ or outcome assessment/ or outcomes research/ or medical assessment/ or measurement/ or exp measurement precision/ or exp measurement accuracy/ or measurement error/ or exp systematic error/ or exp performance measurement system/ or exp measurement repeatability/ or intermethod comparison/ or data collection method/ or system analysis/ or validation study/ or feasibility study/ or exp quality control/ or rating scale/ or scoring system/ or summated rating scale/ or qualitative analysis/ or quantitative analysis/ or correlation analysis/ or "constants and coefficients"/ or correlation coefficient/ or cronbach alpha coefficient/ or kappa statistics/ or correlation function/ or exp reliability/ or discriminant analysis/ or exp validity/ or valid*.hw. or factorial analysis/ or observer variation/ or psychometry/ or (audit or audits or psychometr* or clin?metr* or ((outcome* or clinical or observer* or utility or satisfaction or QoL or quality of life or score or scores or method or methods or physicians or dermatologists or modelling or objective) adj3 assessm*) or clinical asses* or outcome measure* or observer variation* or reproducib* or reliab* or unreliab* or valid* or coefficient or homogeneity or homogeneous or ((internal or external) adj3 (consistency or inconsistency)) or cronbach* or (item and (correlation* or selection* or reduction*)) or ((item or items) adj3 (discriminant* or convergent* or divergent*)) or agreement or precision or imprecision or (precise adj values) or (test and retest) or accuracy test* or stability or interrater or intrarater or intertester or intratester or interobserver or intraobserver or intertechnician or intratechnician or interexaminer or intraexaminer or interassay or intraassay or interindividual or intraindividual or interparticipant or intraparticipant or ((inter or intra) adj (rater or tester or observer or technician or examiner or assay or individual or participant)) or kappa or kappa's or kappas or repeatab* or ((replicab* or repeated) and (measure or measures or findings or result or results or test or tests)) or generaliza* or generalisa* or concordance or (intraclass and correlation*) or discriminative or (known adj group) or (factor adj (analy* or structure*)) or dimension* or interscale or inter-scale or interscales or inter-scales or subscale* or sub-scale* or ((multitrait* or multi-trait*) and (scaling or scale*)) or error or errors or ((individual or interval or rate) adj variability) or (variability adj5 (analy* or values)) or (uncertainty and (measurement or measuring)) or sensitiv* or responsive* or ((limit or limits) and detection) or ((minim* or lowest) adj2 detectable adj2 (concentration* or dose* or level* or amount*)) or interpretab* or (small* and (real or detectable) and (change or difference)) or meaningful change* or ((minimal* or minimum) adj2 (meaningful or important or detectable or real or identifiable or relevant) adj3 (change* or difference* or improvement*)) or ((minimal or minimally or clinical or clinically) and (important or significant) and (change* or difference* or improvement*)) or (MDC adj2 value*) or MCID or MCIDs or MICD or MICDs or MCII or MCIC or MCICs or ((ceiling or floor) adj2 effect*) or item response* or IRT or rasch or ((differential or fit) adj2 item*) or DIF or computer adaptive test* or item bank* or cross-cultural equivalen*).tw,ot,kw.

**Appendix 4: Study characteristics**

| **Primary author** | **Year** | **Medical Center** | **Country** | **Study design** | **No. of patients** | **Patient Age  (if possible: mean (range;**  **median))** | **Female:**  **male ratio** | **Size CMN** | **Location CMN** | **Classification system used** | **Aim**  **Intervention used** | **Mean follow-up (range)** | **Level of evidence** |
| --- | --- | --- | --- | --- | --- | --- | --- | --- | --- | --- | --- | --- | --- |
| AL Mutairi H. et al.(1) | 2020 | Multicenter | Saudi-Arabia | Systematic Review | 595 | Unreported | Unreported | small to giant CMN | Unreported | PAS | Evaluation of treatment  Watchful waiting  Surgical intervention  (excision, serial excision, curettage, tissue expander, dermabrasion)  Laser | Unreported | 3- |
| Alkureishi L. et al. | 2018 | Shriners Hospital for Children Chicago | USA | Retrospective | 19 | Unreported | Unreported | Unreported | Unreported | Unreported | Evaluation of treatment  Surgical intervention  (Excision with reconstruction with skin flap) | 14 years (range 2 to 21 years) | 3 |
| Ben-Ari A. et al. | 2020 | Hadassah Medical Center | Israel | Prospective | 30 | Range 1 to 13 years (mean 4.23 years, median 3.1 years) | 1,1:1 | Unreported | Back, head, abdomen, buttocks and other (knee, groin, arm, shoulder, blades, hand) | Unreported | Post-traumatic stress symptoms evaluation  Surgical intervention (not specified) | 4 months | 3 |
| Calbet-Llopart M. et al. | 2020 | Multi center | Spain, France | Prospective | 166 | (mean 16.81 years, median 16.54 years) | 1,63:1 | medium to giant CMN | Head, Head and Trunk, Extremities | Krengel: medium to giant CMN, 6B: giant CMN only | Association of MC1R variants with CMN characteristics  Unreported | Unreported | 3 |
| Carmen Ceballos-Rodríguez, M. et al. | 2021 | La Paz University Hospital - Madrid | Spain | Retrospective | 136 | Range 1 month to 59 years; median 9 years | 1,56 | giant CMN | Cranofacial, Extremities, Trunk | PAS/6B | Evaluation of treatment  Surgical intervention (Excision) | Unreported | 4 |
| Cho H. et al. | 2019 | Hanyang University Seoul Hospital | South-Korea | Prospective | 15 | Range 13 to 73 years (mean 26.0 years) | 02:01 | small to medium kissing nevus | eyelids | PAS | Evaluation of treatment  CO2 Laser | 10 months (range 3–19 months) | 4 |
| Elmelegy N. et al. | 2020 | Tanta University - Gharbia | Egypt | Prospective | 25 | Range 2 to 45 years (mean 18 years) | 1,5:1 | small to medium CMN | Nasal and perinasal region | PAS | Evaluation of treatment  Carbon dioxide cryotherapy | 1.0 year (range 6 months to 1.5 years) | 4 |
| Fahradyan A. et al. | 2019 | Multicenter | USA | Retrospective | 32 | Range 3.3 months to 15.8 years (mean 4.4 years; median 4.5 years) | 1,46:1 | small to giant CMN | Face (eyebrows, eyelid margins, nasal alae) | Krengel (PAS) | Estimate prevalence of melanoma  Surgical intervention | 5.6 years (range 1.0 to 14.4 years) | 3 |
| Funayama E. et al. | 2019 | Hokkaido University Hospital | Japan | Prospective | 19 | Range 1 month to 6 years (median 9 months) | Unreported | medium CMN | head, neck, trunk, limb | Unreported | Evaluation of treatment  Laser  (PDL & QSRL) | 1 year | 4 |
| Gu Y. et al. | 2019 | Shanghai Ninth People's Hospital | China | Prospective | 20 | Unreported | 2,3:1 | Unreported | Face (eyelid, peri-orbital region) | Unreported | Evaluation of treatment  Laser (CO2/ER:YAG/combination) | 20.5 months (range 6 to 48 months) | 4 |
| Han J. W. et al. | 2021 | Inje University School of Medicine | Korea | Retrospective | 78 (42 anchoring/36 conventional) | n=42 (mean 14.2 years, SD 3.7 years) n=36 ( mean 17.6 years, SD 4.7 years) | Unreported | small to large CMN | Face, Arm, Leg, Back, Abdomen | Unreported | Evaluation of treatment  Surgical intervention  (de-epithelialized dermal flaps) | n=42 (mean 12.2 months, SD 2.4 months) n=36 (mean 14.8 months, SD 4.7 months) | 3 |
| Hong S. et al. | 2019 | National Hospital of Dermatology and Venereology and Saint Paul Hospital | Vietnam | Prospective | 20 | Unreported | Unreported | giant CMN | Unreported | Unreported | Evaluation of treatment  Surgical intervention  (Excision) | 6.0 months | 4 |
| Kim J. et al. | 2019 | Ajou University Hospital | South-Korea | Prospective - observational | 100 | Range 10 months to 103 months | 1,7:1 | area before resection (in cm2) & long diameter (in cm) | trunk, face, scalp, extremities | Total Body Surface Area (TBSA) and long diameter | Evaluation of treatment  Surgical intervention  (Excision) | Unreported | 4 |
| Kim M. J. et al. | 2020 | Ajou University School of Medicine | Korea | Retrospective | 55 (24 single/31 serial) | mean 6.59 years, SD 7.76 years | 1,38 | giant CMN | Head, Lower extremity, Upper extremity, Back, Trunk | Unreported | Evaluation of treatment  Surgical intervention  (Excision) | Mean 23.74 months, SD 28.20 months | 4 |
| Malladi N. et al. | 2020 | KEM Hospital - Mumbai | India | Cross-sectional | 44 | Range 0 to 20 years | unspecified | Unreported | Unreported | Unreported | Evaluation of Clinical, epidemiological and dermoscopic patterns  Unreported | no follow-up | 4 |
| Masnari O. et al. | 2019 | University Children's Hospital - Zurich | Switzerland | Cross-sectional | 235 | Range 1 month to 18 years (mean 6.3 years; median 5.0 years) | 0,93:1 | all sizes CMN | face, scalp, neck, collar, arms/ shoulders, hands, chest, abdomen/flank, back/buttocks, genitals,  and legs/feet | Total Body Surface Area (TBSA) | Evaluation of health-related quality of life and psychological  adjustment  Surgical intervention  (Excision) | Unreported | 3 |
| Mosa A. et al. | 2019 | Hospital for Sick Children - Toronto | Canada | Prospective | 30 | Range 1 to 25 years (mean 9.2 years, median 10 years) | 1,73:1 | small to large CMN | Head and neck, trunk, extremities | Unreported | Perspectives and expectations  of patients and families  Unreported | Unreported | 3 |
| Neuhaus K. et al. | 2020 | University Children's Hospital - Zurich | Switzerland | Prospective | 163 | Range 4 to 18 years | 1,46:1 | Unreported | face, scalp, neck, collar, arms/shoulders, hands, chest, abdomen/ flank, back/buttocks, genitals, legs/feet | Total Body Surface Area (TBSA) | Skin-related Quality of Life  Surgical intervention  (Excision) | Unreported | 3 |
| Neuhaus K. et al. | 2020 | University Children's Hospital Zurich | Switzerland | Retrospective | 44 patients (self-report) and 249 (proxy-report) | n=44 Range 14 to 24 years (mean 17.50 years, median 2.25 years) n=246 Range 0-18 years (mean 6.34 years, median 5.08 years) | 4,5:1 and 0,98:1 | All sizes | Face, Scalp, Neck, Collar, Arms/shoulders, Hands, Chest, Abdomen, Back, Legs/feet, Genitals | Total Body Surface Area (TBSA) | Treatment evaluation  Surgical intervention  (Excision) | unreported | 4 |
| Oh Y. et al. | 2019 | Severance Hospital and Gangnam Severance Hospital | South-Korea | Retrospective | 67 | (mean 13.42 years) | 2,04:1 | mean 36.57 cm² | face, trunk, extremities | Unreported | Treatment evaluation  Laser only group (ablative laser/ pigment specific laser)  Combination group (partial excision with laser) | 3.40 years | 3 |
| Pellino G. et al. | 2020 | Multicenter | Italy | Systematic Review | 24 | Range 12 to 322 months (median 5 months) | 01:02 | giant CMN | Unreported | PAS | Evaluation of neurological symptoms  Parenchymal neurocutaneous melanosis specific interventions  (anti-epileptic drug [AED] therapy or surgical epilepsy therapy) | 50 months (range 6 to 286 months) | 3- |
| Polubothu S. et al. | 2020 | Great Ormond St. Hospital - London | United Kingdom | longitudinal cohort - prospective | 110 | Range 0.0 to 17.4 years | 1,62:1 | all sizes | Unreported | PAS | CMN lightening over time  Watchful waiting  Surgical intervention  (superficial removal techniques) | Mean 5.3 years ( range 0.2-16.0 years) | 3 |
| Qiao C. et al. | 2019 | Shanghai Ninth People's Hospital | China | Retrospective | 35 | Range 3 to 36 years (mean 20.4 years; median 8.4 years) | 3,38:1 | medium CMN | Unreported | PAS | Evaluation of treatment  Surgical intervention  (vertical/near vertical linear closure) | 1 year | 4 |
| Roh D. et al. | 2020 | Pusan National University Hospital | South-Korea | Retrospective | 43 | (mean 15.9 years; median 14.6 years) | 1,18:1 | small to medium CMN | palm/sole | Unreported | Evaluate difference between acquired and congenital melanocytic naevi  Unreported | Unreported | 3 |
| Rouillé T. et al. | 2019 | Unreported | France | Prospective | 17 | Range 4 months to 8 years (median 18 months) | 2,4:1 | large to giant CMN | head, trunk, arm, leg | Krengel (PAS) | Evaluation of treatment  MEK-inhibitors  AKT-inhibitors | Unreported | 4 |
| Sakhiyah J. et al. | 2019 | New Civil Hospital - Asarwa | India | Retrospective | 17 | Range 22 to 52 years (mean 31.7 years) | 7,5:1 | small CMN | Face | Krengel | Evaluation of treatment  Carbon dioxide laser ablation performed by a surgeon | Unreported | 4 |
| Tomás-Velasqués A. et al. | 2020 | Multi center | Spain | exploratory case control - Prospective | 24 | (mean 5.7 years; median 3.4 years) | 03:01 | medium to giant CMN | Unreported | Krengel (PAS) | Level of S-100B  Unreported | Unreported | 3 |
| Wu M. et al. | 2020 | Shanghai Ninth People's Hospital | China | Retrospective | 98 | Range 0.25 - 37 years (median 6 years) | 0,72:1 | giant CMN | Head and Neck, Trunk, Extremities | PAS | Histopathological characteristics  Unreported | Unreported | 3 |
| Zalaudek I. et al. | 2020 | Multi center | Austria and Italy | Retrospective | 47 | (mean 39.02 years; median 17.63 years) | 1,04:1 | (15.13±21.33 mm) C-NAM | Unreported | Unreported | Histopathological characteristics  Unreported | Unreported | 3 |

**Appendix 4: COSMIN Risk of Bias checklist**

Score: V= very good; A = adequate; D = doubtful; I = inadequate; N= not applicable


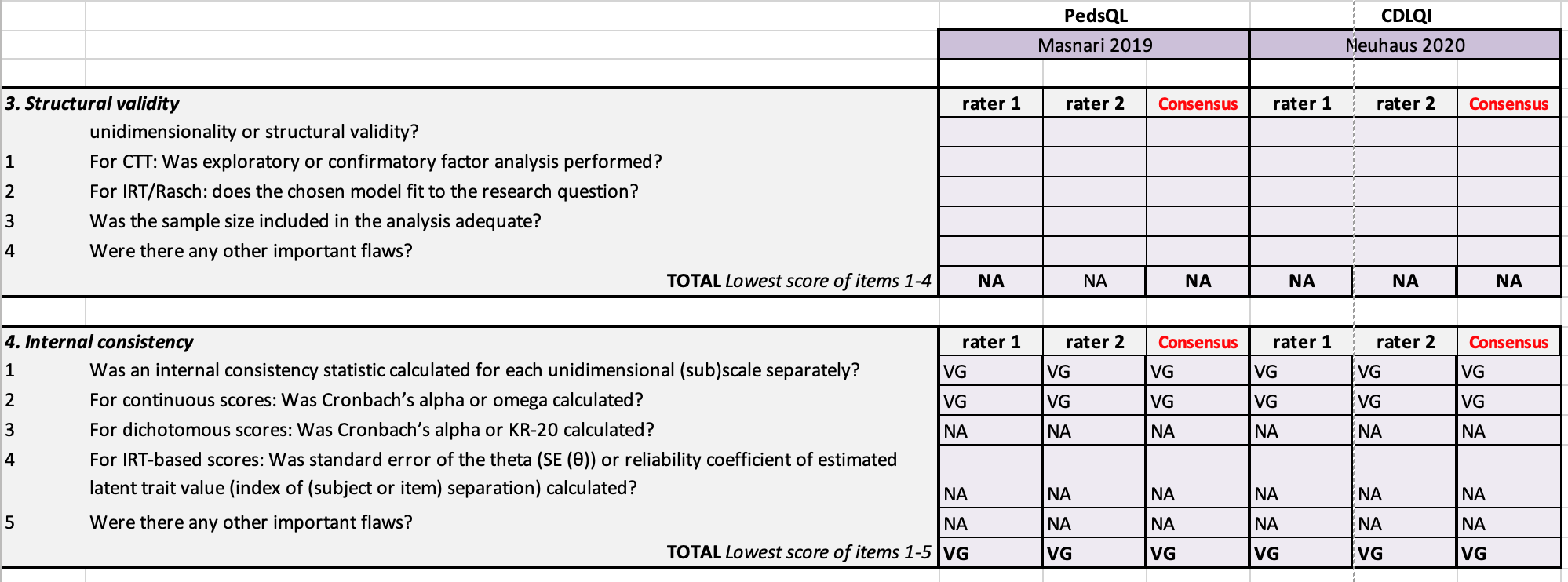


We present the risk of bias scores of structural validity and internal consistency. Structural validity was not evaluated, what influences the rating of internal consistency (shown in Table 4). Like structural validly, all other measurement properties were not evaluated by the two included studies.
